# Unveiling the causal link between metabolic factors and ovarian cancer risk using Mendelian randomization analysis

**DOI:** 10.3389/fendo.2024.1401648

**Published:** 2024-06-05

**Authors:** Li Han, Shuling Xu, Dongqi Zhou, Rumeng Chen, Yining Ding, Mengling Zhang, Meihua Bao, Binsheng He, Sen Li

**Affiliations:** ^1^ Hunan Provincial University Key Laboratory of the Fundamental and Clinical Research on Functional Nucleic Acid, Changsha Medical University, Changsha, China; ^2^ Hunan Key Laboratory of The Research and Development of Novel Pharmaceutical Preparations, School of Pharmaceutical Science, Changsha Medical University, Changsha, China; ^3^ School of Life Sciences, Beijing University of Chinese Medicine, Beijing, China; ^4^ Department of Traditional Chinese Medicine, Sichuan Taikang Hospital, Chengdu, Sichuan, China; ^5^ School of Stomatology, Changsha Medical University, Changsha, China; ^6^ The Hunan Provincial Key Laboratory of the TCM Agricultural Biogenomics, Changsha Medical University, Changsha, China

**Keywords:** ovarian cancer, metabolic factors, risk factors, Mendelian randomization, causal association

## Abstract

**Background:**

Metabolic abnormalities are closely tied to the development of ovarian cancer (OC), yet the relationship between anthropometric indicators as risk indicators for metabolic abnormalities and OC lacks consistency.

**Method:**

The Mendelian randomization (MR) approach is a widely used methodology for determining causal relationships. Our study employed summary statistics from the genome-wide association studies (GWAS), and we used inverse variance weighting (IVW) together with MR-Egger and weighted median (WM) supplementary analyses to assess causal relationships between exposure and outcome. Furthermore, additional sensitivity studies, such as leave-one-out analyses and MR-PRESSO were used to assess the stability of the associations.

**Result:**

The IVW findings demonstrated a causal associations between 10 metabolic factors and an increased risk of OC. Including “Basal metabolic rate” (OR= 1.24, *P*= 6.86×10^-4^); “Body fat percentage” (OR= 1.22, *P*= 8.20×10^-3^); “Hip circumference” (OR= 1.20, *P*= 5.92×10^-4^); “Trunk fat mass” (OR= 1.15, *P*= 1.03×10^-2^); “Trunk fat percentage” (OR= 1.25, *P*= 8.55×10^-4^); “Waist circumference” (OR= 1.23, *P*= 3.28×10^-3^); “Weight” (OR= 1.21, *P*= 9.82×10^-4^); “Whole body fat mass” (OR= 1.21, *P*= 4.90×10^-4^); “Whole body fat-free mass” (OR= 1.19, *P*= 4.11×10^-3^) and “Whole body water mass” (OR= 1.21, *P*= 1.85×10^-3^).

**Conclusion:**

Several metabolic markers linked to altered fat accumulation and distribution are significantly associated with an increased risk of OC.

## Introduction

Ovarian cancer (OC) is one of the leading causes of gynecological cancer-related death among females globally, ranking as the fifth most common reason for cancer-related mortality, with a five-year survival rate of less than 29% (1, 2). According to the most recent estimates from 2017, this condition affects around 224,940 females globally (3). Experts predict a 55% increase in OC prevalence and 67% increase in mortality rates by 2035 (4). Furthermore, more than 90% of the earliest malignant ovarian tumors are epithelial in origin (5). As a result, early detection of OC is critical in lowering the high fatality rate among women.

Metabolic processes play an important role in various diseases (6–10), and the investigation into the impact of metabolic and anthropometric factors on OC is becoming a progressively appealing research area (11–14). The bulk of OC is related to the development of ascites (15). Malignant ascites forms a distinct tumor microenvironment with several metabolic regulators, including growth factors, chemokines, and cytokines, which promote the invasion and resistance to medications in various types of cancers (3, 16–20). Furthermore, OC cells need adipocytes for energy, which might affect lipid metabolism and serum levels (21, 22). Multiple studies have indicated that overweight and obesity elevate the likelihood of developing OC (23–25). For instance, a meta-analysis and systematic review revealed that high body mass index (BMI) was associated with an increased risk of OC (14). Additionally, a prospective investigation found that for every 10-centimeter increase in waist circumference, the pooled relative risk (RR) for OC was 1.06 (26). However, one study showed no evidence connecting waist circumference with an increased chance of developing OC (27). Anthropometric measurements, such as hip circumference and waist-to-hip ratio, are considered risk factors for metabolic disorders, however evidence on their relationship with OC is inconsistent (27–30). Given the lack of reliable and consistent evidence, more research is needed to determine the overall risk and histological associations between numerous metabolic factors and OC.

We used Mendelian randomization (MR) to evaluate the causal relationship between numerous metabolic factors and OC. MR methods successfully reduce reverse causation and residual confounding by utilizing instrumental variables (IVs) that are closely related to the exposure (31). Our goal is to improve understanding of OC risk factors and develop new prevention strategies.

## Methods

### Study design

This study used IVs and MR analysis to emulate the randomization procedure in a randomized controlled experiment. It focused on several elements: 1) IVs were significantly associated with exposures; 2) The causal link between exposures and outcomes was assessed using inverse variance weighting (IVW), MR-Egger, and weighted median estimate (WM); 3) Sensitivity analyses were used to determine the robustness of the findings.

### Data sources

This study used data solely from public databases. The research involved a group of European people. As exposure indicators, a complete collection of 23 anthropometric and metabolic parameters was used. **Supplementary Table 1** provides further information on these 23 exposure variables. OC was identified as research outcome, and the information of genome-wide association study (GWAS) data is also given in **Supplementary Table 1**.

### Selection of IVs

The selection criteria included *P* < 5×10^-8^, which identified IVs with substantial relationships with the exposure variables. The clump=TRUE option was used to eliminate linkage disequilibrium (LD) and improved the accuracy of the IVs, as described in our previous publications (32–35). The parameters of r^2^ = 0.001 and kb=10000 represented the LD threshold and distance for clumping, respectively. To summarize, r^2^ = 1 implied a perfect LD association between two single nucleotide polymorphisms (SNPs), whereas r^2^ = 0 showed complete LD equilibrium, implying that the distribution of these two SNPs is random. The parameter kb denotes the length of the region evaluated for LD. In genetics, it is widely assumed that linked genetic loci on a chromosome are frequently inherited together. As a result, in our investigation, we utilize r^2^ = 0.001 and kb=10000 to achieve more accurate results while taking into consideration probable LD. We also calculated the F statistic: a high F statistic (greater than or equal to 10) indicated a strong IV (36).

### MR analysis

The IVW approach was the principal analytic technique employed in this work. The WM technique is more resistant to some invalid IVs. Hence, it is employed as a complement to the IVW approach in this study (37). The MR-Egger method serves as an additional strategy, employing the intercept term to investigate potential pleiotropic effects (38).

### Sensitivity analysis

LDlink was used to assess the relationship between all exposure-associated IVs and potential confounders (39, 40), and sensitivity analysis was performed after excluding these SNPs with external pleiotropy. The study used pleiotropy-corrected data from MR-PRESSO to indicate any likely deviants in the analysis. The Cochrane Q statistic was used to assess the heterogeneity present. Furthermore, a detailed sensitivity analysis using a leave-one-out technique was performed to check the reliability of the findings and to assess the influence of each IV on the causal relationship. Given the binary character of the outcome, the MR evaluation used odds ratios (ORs), as well as 95% confidence intervals (CIs), to present causal effect. To address the issue of multiple testing, the 5% false discovery rate (FDR) was set. All MR assessments were carried out utilizing the TwoSampleMR package in the R programming environment.

## Results

### The F-statistics of IVs

This research examined a total of 23 metabolic and anthropometric variables. 10 traits played a causal influence in OC. The F-statistic values for the associated IVs of these 10 variables were all more than 29, indicating good IVs (**Supplementary Table 2**).

### MR analysis results

**Figure 1:**
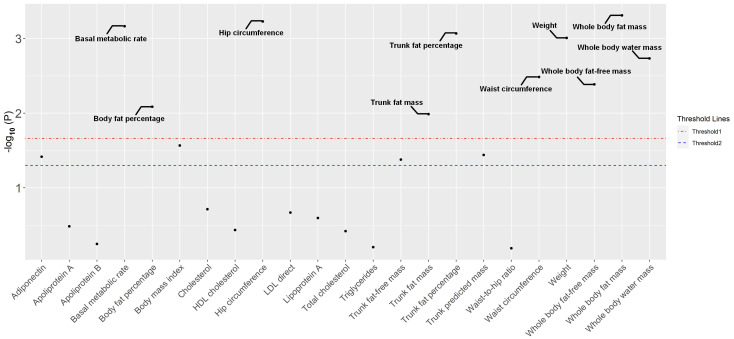
The distribution of *P*-values for the associations between 23 metabolic factors related to anthropometric indicators and ovarian cancer in the MR analysis. Two thresholds were used to assess significance. One represented the threshold adjusted for false discovery rate (threshold line 1), while the other relied on a commonly used *P*-value (0.05). MR, Mendelian randomization.

**Figure 2:**
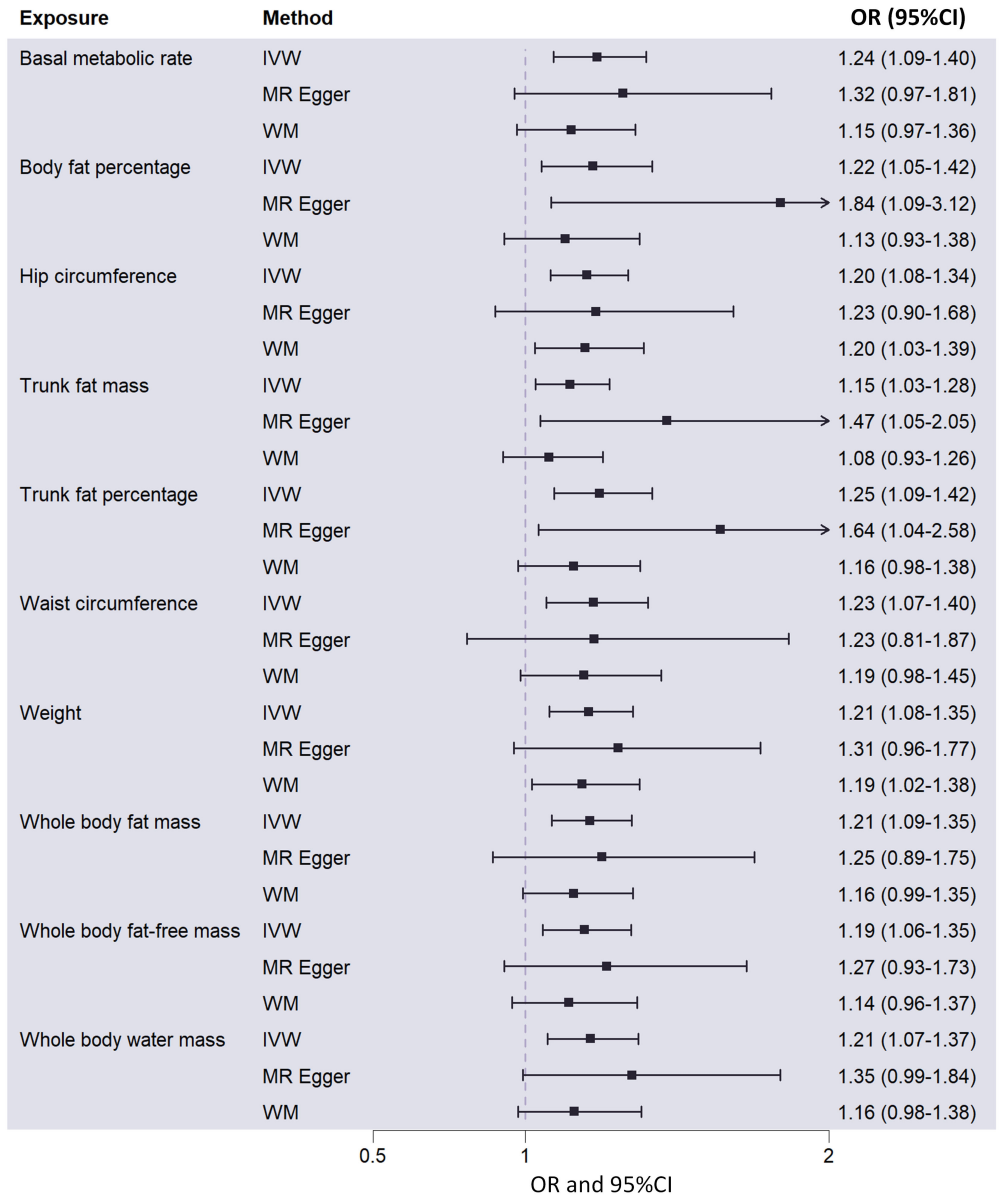
Causal effects of 10 metabolic factors on ovarian cancer in the MR analysis, showing odds ratios and corresponding 95% confidence intervals. MR, Mendelian randomization; IVW, inverse-variance weighted; WM, weighted median; OR, odd ratio; 95%CI, 95% confidence intervals.

**Figure 3:**
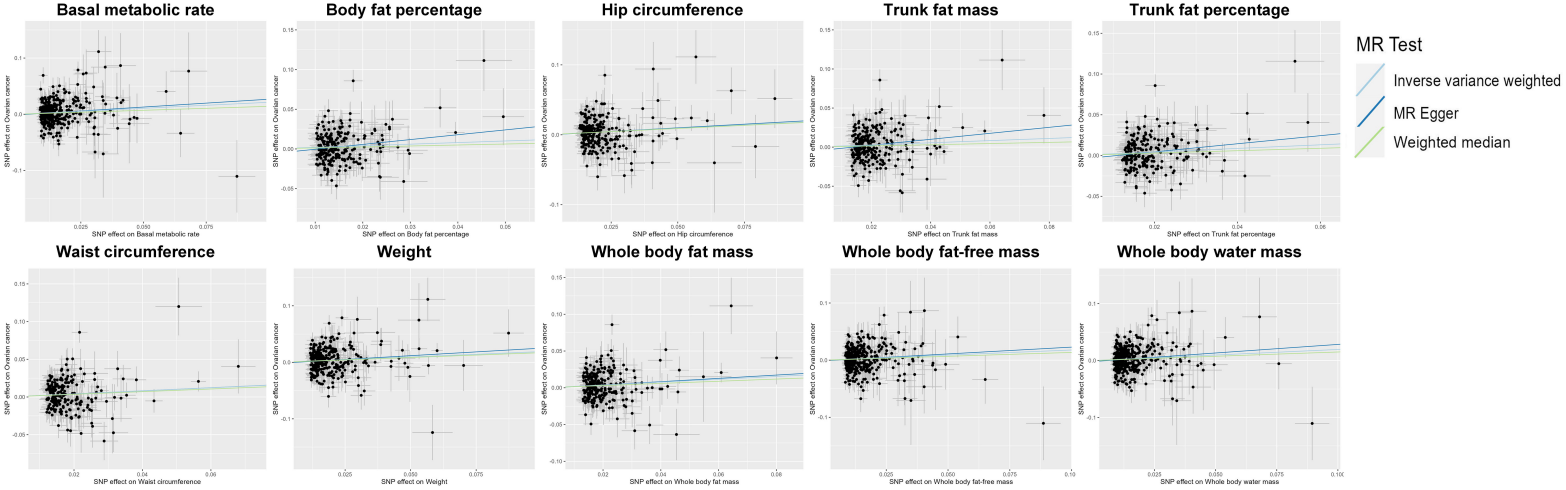
Scatter plot indicating the causal associations between 10 metabolic factors and ovarian cancer. SNP, single nucleotide polymorphism; MR, Mendelian randomization.

Among the 23 metabolic and anthropometric variables, 10 indicated a causative association with OC (**Figure 1**). The IVW results of the MR analysis for each of the 10 traits were listed below: “Basal metabolic rate - OC” (OR: 1.24; 95% CI: 1.09,1.40), “Body fat percentage - OC” (OR: 1.22; 95% CI: 1.05,1.42), “Hip circumference - OC” (OR: 1.20; 95% CI: 1.08,1.34), “Trunk fat mass - OC” (OR: 1.15; 95% CI: 1.03,1.28), “Trunk fat percentage - OC” (OR: 1.25; 95% CI: 1.09,1.42), “Waist circumference - OC” (OR: 1.23; 95% CI: 1.07,1.40), “Weight - OC” (OR: 1.21; 95% CI: 1.08,1.35), “Whole body fat mass - OC” (OR: 1.21; 95% CI: 1.09,1.35), “Whole body fat-free mass - OC” (OR: 1.19; 95% CI: 1.06,1.35), “Whole body water mass - OC” (OR: 1.21; 95% CI: 1.07,1.37) (**Figure 2**). In conclusion, all 10 metabolic factors were positively causally associated to OC(**Figure 3** and **Supplementary Table 3**). After excluding IVs that were associated with potential confounders (e.g., smoking status and alcohol consumption), the majority of our analyses remained unchanged (**Supplementary Table 4**). Reverse MR analysis of OC as the exposure and traits as outcomes revealed no evidence of reverse causality (all *P* > 0.05) (see **Supplementary Table 5**).

### Results of the sensitivity analysis

**Figure 4:**
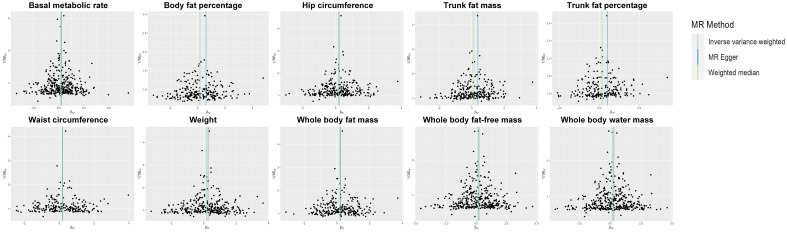
Funnel plot of the IVs. IV, instrumental variable; SE, standard error; MR, Mendelian randomization

The Cochrane Q statistic was used to evaluate the existence of heterogeneity (**Supplementary Table 6**), and the funnel plots demonstrated that the distribution of IVs exhibited symmetry (**Figure 4**). The MR-Egger test showed no significant horizontal pleiotropy (all *P >*0.05), implying that the data are reliable (see **Supplementary Table 7**). **Supplementary Table 8** shows the 10 traits remained associated with OC at suggestive evidence of significance after correcting for outliers (all *P <*0.05). **Supplementary Figure 1** depicts the findings of the leave-one-out analysis, which showed that the majority of SNPs did not cross the null line after being removed, indicating that the study had low potential bias.

## Discussion

Obesity and an increased risk of OC have been extensively studied (41, 42), but the impact of body measurement markers in multiple body areas on OC risk should also be investigated. Our MR analysis demonstrated that, after FDR correction, 10 metabolic parameters still had significant positive causal relationships with OC out of the total of 23 metabolic and anthropometric factors studied. These continuous variables are related to body fat distribution, including whole body fat mass, basal metabolic rate (BMR), whole body fat-free mass, and waist circumference.

Research indicates that varying body fat distribution might impact the progression of OC. A longitudinal research found that women with higher fat mass had a significantly increased chance of developing OC (43). An MR investigation found a connection between BMR and higher OC risk (44). Body composition is a better predictor of overall health status in OC patients than weight or BMI (45). Another MR study discovered that the proportion of trunk fat is a surrogate for abdominal adiposity, indicating a causal positive relationship with OC (46). In a study of normal-weight individuals (40-70 years old) in the UK Biobank cohort, researchers found no correlation between waist circumference, trunk fat mass index, trunk fat mass ratio, and waist-hip ratio with the risk of OC in women (47). The complicated influence of numerous anthropometric parameters on OC necessitates more exploration. Our findings may provide additional causal insights into the effect of fat distribution on OC.

The distribution of body fat in humans might correlate with leptin levels, which play a crucial role in lipid metabolism (48). Research indicates that leptin plays a role in determining the resting metabolic rate in lean individuals (49). A prospective study in Sweden analyzed serum samples to examine leptin concentrations and their correlation with weight history (50). The study revealed that elevated leptin levels posed a risk for future weight gain in women aged 38 to 46 (50). Additionally, a four-year longitudinal study carried out in America identified that heightened plasma leptin levels in overweight males could signify leptin resistance and eventual weight gain (51). A longitudinal study involving African Americans revealed a distinct, independent association between heightened hip circumference, expanded waist circumference, and elevated serum leptin levels (52). Moreover, a recent study in young adults aged 20 to 21 demonstrated a positive correlation between serum leptin levels, waist circumference, and body fat percentage in both men and women (53). Furthermore, a study investigating the potential relationship between trunk fat levels and breast gene expression revealed that increased trunk fat levels were correlated with higher levels of leptin (54). These findings suggest that leptin may play a role in human fat distribution and endocrine metabolism. Leptin is synthesized and released by adipocytes to interact with its specific receptor, the leptin receptor (LEP-R), located in white adipose tissue (55). LEP-R facilitates the diverse effects of leptin and plays a vital role in regulating body weight. Leptin resistance is typified by diminished satiety, increased nutrient intake, and subsequent weight gain, ultimately contributing to the development of obesity (56).

Adipose tissue levels in the blood increase sensitivity to leptin expression, which might explain the link between obesity and the risk of OC (57). More than half of patients with epithelial ovarian cancer (EOC) in the Middle East demonstrated overexpression of leptin and its receptors, leading in a shorter overall survival (58). The advancement of OC under the influence of leptin is associated with the phosphorylation of STAT3 and the estrogen receptor (ER), as well as the synthesis of ER-responsive genes, which impacts the overall longevity of OC patients (59–61). Anomalously activated STAT3 promotes uncontrolled tumor cell growth and survival by a number of ways, including increased production of oncogenes like Myc proto-oncogene protein (c-myc) and cyclin D, as well as anti-apoptotic proteins like B-cell lymphoma-extra large (Bcl-XL) and Myeloid cell leukemia 1 (MCL-1) (62, 63). Furthermore, leptin boosts the circulating levels of follicle-stimulating hormone (FSH), which is essential for OC cell activation and proliferation (64). Hence, it is speculated that the 10 metabolic factors related to fat distribution identified in this study may contribute to the pathogenesis of OC through the overexpression of leptin and its receptor.

Hypoxia Inducible factor-1 alpha (HIF-1α), which can be induced by hypoxia and many other factors (65), regulates gene transcription in tumor cells and is corelated with the tolerance of chemotherapy (66–68). HIF-1α can be activated by hypoxia in obesity-related adipose tissue, and HIF-1α promotes the expression of collagen remodeling genes such Collagen type I alpha 1 chain (COL1A1) and Lysyl oxidase (LOX) in ovarian surface epithelial cells and activates Macrophage Type 2 (M2) macrophages (69–72). Surface epithelial cell collagen remodeling and M2 macrophages are associated with a bad outcome in OC patients (70). Adipocytes undergo phenotypic changes when they come into contact with tumor cells, becoming cancer-associated adipocytes (CAAs) and enduring delipidation at the leading edge of tumor invasion, adopting a fibroblast-like phenotype (73). CAAs can effectively halt the cell cycle, elevate the expression of genes linked to cell cycle arrest, and reduce the expression of genes that facilitate cell growth. Additionally, the transformation from regular adipocytes to CAAs may involve cellular aging (74). This process involves an increase in inflaming cytokine release (Interleukin-6 (IL-6) and Plasminogen activator inhibitor-1 (PAI-1)) and may result in the transformation of non-malignant stromal cell types (fibroblasts and macrophages) into cancer-related fibroblasts and macrophages (12, 41, 75, 76). Moreover, CAAs also secrete leptin, a hormone known to influence the immune system, potentially facilitating tumor metastasis and immune evasion (77). The role of a relationship between the development of non-malignant stromal cells and mature adipocytes is currently unknown and warrants more research.

Increased levels of androgens and estrogens, decreased progesterone, and control over the insulin-like growth factor (IGF) axis may be the molecular processes behind the link between fat accumulation and the risk of OC (78). Furthermore, obesity and fat redistribution, particularly in the central region, are known to enhance cancer susceptibility (79–83). Nonetheless, the mechanisms behind the relationship between body fat measurements and the risk of OC in adults of normal weight are still not fully understood.

Our research has various advantages. Our study examines the impact of many metabolic factors on OC by MR analysis, and the identified causal relationships are clinically significant. Furthermore, rigorous sensitivity studies confirmed the reliability and stability of our conclusions. Finally, by including genetic variants, we decreased confounding interference and so preserved the study’s validity.

Nonetheless, our study has limitations. To begin, the risk of selection bias cannot be fully ruled out. Furthermore, While the MR-Egger intercept test did not indicate the presence of horizontal pleiotropy, it is important to note that the possibility of its existence cannot be entirely ruled out. Moreover, because our study only included Europeans, the applicability of our findings to other ethnic groups may be restricted. Future research in diverse ethnic populations, including Asians and Africans, is warranted. Finally, the effect of metabolic factors on OC may not be a simple linear relationship, which cannot be evaluated by our study.

## Conclusion

We conducted a comprehensive investigation of the relationships between metabolic indicators and OC risk. We discovered that fat accumulation and distribution contribute to metabolic alterations that have a deleterious impact on OC. The results will also potentially accelerate the identification of indicators and assessment methods, thus enhancing early intervention and treatment strategies for OC. Additional comprehensive studies are necessary to clarify the underlying mechanisms of our observations.

## Data availability statement

The original contributions presented in the study are included in the article/**Supplementary Material**. Further inquiries can be directed to the corresponding authors.

## Author contributions

LH: Writing – original draft. SX: Writing – original draft. DZ: Writing – original draft. RC: Writing – original draft. YD: Writing – original draft. MZ: Writing – original draft. MB: Writing – review & editing. BH: Writing – review & editing. SL: Writing – review & editing.
