# Supplementary material for: Unveiling the causal link between metabolic factors and ovarian cancer risk using Mendelian randomization analysis
Source: Front Endocrinol (Lausanne). 2024 Jun 5;15:1401648. doi: 10.3389/fendo.2024.1401648 (PMC11185996; doi:10.3389/fendo.2024.1401648)
Supplement: Supplementary file 1 [file DataSheet_1.docx]

Supplementary Table 1. Information of GWAS summary datasets used in MR analyses

| Traits | GWAS.ID |
| --- | --- |
| Ovarian cancer | ieu-a-1120 |
| Adiponectin | ieu-a-1 |
| Apoliprotein A | ukb-d-30630_irnt |
| Apoliprotein B | ukb-d-30640_irnt |
| Basal metabolic rate | ukb-a-268 |
| Body fat percentage | ukb-a-264 |
| Body mass index | ieu-b-40 |
| Cholesterol | ukb-d-30690_irnt |
| HDL cholesterol | ukb-d-30760_irnt |
| Hip circumference | ukb-a-388 |
| LDL direct | ukb-d-30780_irnt |
| Lipoprotein A | ukb-d-30790_irnt |
| Total cholesterol | ieu-a-301 |
| Triglycerides | ukb-d-30870_irnt |
| Trunk fat mass | ukb-a-291 |
| Trunk fat percentage | ukb-a-290 |
| Trunk fat-free mass | ukb-a-292 |
| Trunk predicted mass | ukb-a-293 |
| Waist circumference | ukb-a-382 |
| Waist-to-hip ratio | ieu-a-72 |
| Weight | ukb-a-249 |
| Whole body fat mass | ukb-a-265 |
| Whole body fat-free mass | ukb-a-266 |
| Whole body water mass | ukb-a-267 |
| Abbreviation: GWAS: Genome-wide association study; ID: Identification; MR: Mendelian randomization; ukb: UK Biobank; HDL: High-density lipoprotein; LDL: Low-density lipoprotein. | |

Supplementary Table 2. The F-statistics of IVs

| Exposure | Number of SNPs used as IVs | F-statistic | |
| --- | --- | --- | --- |
|  |  | Min | Max |
| Basal metabolic rate | 348 | 29.90 | 625.06 |
| Body fat percentage | 236 | 30.01 | 433.59 |
| Hip circumference | 261 | 29.77 | 621.27 |
| Trunk fat mass | 261 | 29.79 | 546.41 |
| Trunk fat percentage | 215 | 29.76 | 354.65 |
| Waist circumference | 209 | 29.82 | 660.76 |
| Weight | 308 | 29.73 | 772.58 |
| Whole body fat mass | 258 | 29.75 | 636.20 |
| Whole body fat-free mass | 373 | 29.79 | 588.77 |
| Whole body water mass | 372 | 29.80 | 590.64 |
| Abbreviation: IVs: Instrumental variables; SNP: Single nucleotide polymorphism. | | | |

Supplementary Table 3. MR analysis results

| Expoure | Method | Number of SNPs | OR | 95% CI | *p* |
| --- | --- | --- | --- | --- | --- |
| Basal metabolic rate | IVW | 348 | 1.24 | 1.09~1.40 | 6.86E-04 |
| Basal metabolic rate | MR Egger | 348 | 1.32 | 0.97~1.81 | 8.22E-02 |
| Basal metabolic rate | WM | 348 | 1.15 | 0.97~1.36 | 1.02E-01 |
| Body fat percentage | IVW | 236 | 1.22 | 1.05~1.42 | 8.20E-03 |
| Body fat percentage | MR Egger | 236 | 1.84 | 1.09~3.12 | 2.45E-02 |
| Body fat percentage | WM | 236 | 1.13 | 0.93~1.38 | 2.14E-01 |
| Hip circumference | IVW | 261 | 1.20 | 1.08~1.34 | 5.92E-04 |
| Hip circumference | MR Egger | 261 | 1.23 | 0.90~1.68 | 1.90E-01 |
| Hip circumference | WM | 261 | 1.20 | 1.03~1.39 | 1.76E-02 |
| Trunk fat mass | IVW | 261 | 1.15 | 1.03~1.28 | 1.03E-02 |
| Trunk fat mass | MR Egger | 261 | 1.47 | 1.05~2.05 | 2.59E-02 |
| Trunk fat mass | WM | 261 | 1.08 | 0.93~1.26 | 3.28E-01 |
| Trunk fat percentage | IVW | 215 | 1.25 | 1.09~1.42 | 8.55E-04 |
| Trunk fat percentage | MR Egger | 215 | 1.64 | 1.04~2.58 | 3.28E-02 |
| Trunk fat percentage | WM | 215 | 1.16 | 0.98~1.38 | 9.10E-02 |
| Waist circumference | IVW | 209 | 1.23 | 1.07~1.40 | 3.28E-03 |
| Waist circumference | MR Egger | 209 | 1.23 | 0.81~1.87 | 3.38E-01 |
| Waist circumference | WM | 209 | 1.19 | 0.98~1.45 | 7.24E-02 |
| Weight | IVW | 308 | 1.21 | 1.08~1.35 | 9.82E-04 |
| Weight | MR Egger | 308 | 1.31 | 0.96~1.77 | 8.70E-02 |
| Weight | WM | 308 | 1.19 | 1.02~1.38 | 2.36E-02 |
| Whole body fat mass | IVW | 258 | 1.21 | 1.09~1.35 | 4.90E-04 |
| Whole body fat mass | MR Egger | 258 | 1.25 | 0.89~1.75 | 1.92E-01 |
| Whole body fat mass | WM | 258 | 1.16 | 0.99~1.35 | 6.16E-02 |
| Whole body fat-free mass | IVW | 373 | 1.19 | 1.06~1.35 | 4.11E-03 |
| Whole body fat-free mass | MR Egger | 373 | 1.27 | 0.93~1.73 | 1.33E-01 |
| Whole body fat-free mass | WM | 373 | 1.14 | 0.96~1.37 | 1.39E-01 |
| Whole body water mass | IVW | 372 | 1.21 | 1.07~1.37 | 1.85E-03 |
| Whole body water mass | MR Egger | 372 | 1.35 | 0.99~1.84 | 5.67E-02 |
| Whole body water mass | WM | 372 | 1.16 | 0.98~1.38 | 9.07E-02 |
| Abbreviation: MR: Mendelian randomization; SNP: Single nucleotide polymorphism; CI: Confidence interval; IVW: Inverse-variance weighted; WM: Weighted median | | | | | |

Supplementary Table 4. MR analysis results after removing IVs that were associated with potential confounders

| Expoure | Method | Number of SNPs | OR | 95% CI | *p* |
| --- | --- | --- | --- | --- | --- |
| Basal metabolic rate | IVW | 315 | 1.20 | 1.05~1.37 | 7.63E-03 |
| Body fat percentage | IVW | 207 | 1.18 | 1.01~1.38 | 3.53E-02 |
| Hip circumference | IVW | 223 | 1.16 | 1.03~1.30 | 1.36E-02 |
| Trunk fat mass | IVW | 229 | 1.10 | 0.98~1.23 | 1.08E-01 |
| Trunk fat percentage | IVW | 186 | 1.19 | 1.04~1.36 | 1.14E-02 |
| Waist circumference | IVW | 182 | 1.17 | 1.01~1.36 | 3.58E-02 |
| Weight | IVW | 270 | 1.17 | 1.04~1.32 | 1.18E-02 |
| Whole body fat mass | IVW | 224 | 1.19 | 1.06~1.34 | 2.80E-03 |
| Whole body fat-free mass | IVW | 334 | 1.15 | 1.01~1.32 | 3.36E-02 |
| Whole body water mass | IVW | 331 | 1.17 | 1.02~1.34 | 2.19E-02 |
| Abbreviation: MR: Mendelian randomization; SNP: Single nucleotide polymorphism; CI: Confidence interval; IVW: Inverse-variance weighted | | | | | |

Supplementary Table 5. Reverse MR analysis results

| Outcome | Method | Number of SNPs | Beta | 95%CI | *P* |
| --- | --- | --- | --- | --- | --- |
| Apoliprotein A | IVW | 12 | -0.003 | -0.05~0.04 | 9.12E-01 |
| Apoliprotein B | IVW | 12 | 0.01 | -0.05~0.07 | 7.08E-01 |
| Basal metabolic rate | IVW | 10 | 0.01 | -0.01~0.03 | 5.07E-01 |
| Body fat percentage | IVW | 10 | -0.01 | -0.04~0.03 | 7.03E-01 |
| Cholesterol | IVW | 12 | 0.01 | -0.06~0.09 | 7.29E-01 |
| HDL cholesterol | IVW | 12 | -0.01 | -0.05~0.03 | 6.52E-01 |
| Hip circumference | IVW | 10 | -0.0001 | -0.04~0.04 | 9.94E-01 |
| LDL direct | IVW | 12 | 0.02 | -0.06~0.09 | 6.64E-01 |
| Lipoprotein A | IVW | 12 | 0.001 | -0.01~0.01 | 9.21E-01 |
| Triglycerides | IVW | 12 | 0.002 | -0.03~0.03 | 9.06E-01 |
| Trunk fat mass | IVW | 10 | 0.002 | -0.04~0.05 | 9.32E-01 |
| Trunk fat percentage | IVW | 10 | -0.001 | -0.05~0.04 | 9.78E-01 |
| Trunk fat-free mass | IVW | 10 | 0.01 | -0.01~0.03 | 3.17E-01 |
| Trunk predicted mass | IVW | 10 | 0.01 | -0.01~0.03 | 3.32E-01 |
| Waist circumference | IVW | 10 | -0.02 | -0.06~0.02 | 3.83E-01 |
| Weight | IVW | 10 | 0.003 | -0.03~0.04 | 8.63E-01 |
| Whole body fat mass | IVW | 10 | -0.004 | -0.05~0.04 | 8.53E-01 |
| Whole body fat-free mass | IVW | 10 | 0.01 | -0.01~0.02 | 3.95E-01 |
| Whole body water mass | IVW | 10 | 0.01 | -0.01~0.02 | 4.39E-01 |
| Abbreviation: MR: Mendelian randomization; SNP: Single nucleotide polymorphism; CI: Confidence interval; IVW: Inverse-variance weighted; HDL: High-density lipoprotein; LDL: Low-density lipoprotein. | | | | | |

Supplementary Table 6. Heterogeneity test results

| Exposure | Q | Q_df | Q_*P* |
| --- | --- | --- | --- |
| Basal metabolic rate | 478.58 | 347 | 3.42E-06 |
| Body fat percentage | 321.70 | 235 | 1.46E-04 |
| Hip circumference | 367.89 | 260 | 1.17E-05 |
| Trunk fat mass | 365.17 | 260 | 1.79E-05 |
| Trunk fat percentage | 290.82 | 214 | 3.69E-04 |
| Waist circumference | 295.33 | 208 | 6.45E-05 |
| Weight | 488.02 | 307 | 2.07E-10 |
| Whole body fat mass | 353.87 | 257 | 5.70E-05 |
| Whole body fat-free mass | 496.26 | 372 | 1.64E-05 |
| Whole body water mass | 501.95 | 371 | 6.52E-06 |
| Abbreviation: df: Degree freedom. | | | |

Supplementary Table 7. The pleiotropy of 10 metabolic factors

| Exposure | Egger intercept | *SE* | *P* |
| --- | --- | --- | --- |
| Basal metabolic rate | -0.0011 | 0.003 | 0.65 |
| Body fat percentage | -0.0065 | 0.004 | 0.11 |
| Hip circumference | -0.0005 | 0.003 | 0.88 |
| Trunk fat mass | -0.0053 | 0.004 | 0.13 |
| Trunk fat percentage | -0.0054 | 0.004 | 0.21 |
| Waist circumference | -0.00003 | 0.004 | 0.99 |
| Weight | -0.0016 | 0.003 | 0.59 |
| Whole body fat mass | -0.0007 | 0.003 | 0.84 |
| Whole body fat-free mass | -0.0010 | 0.002 | 0.68 |
| Whole body water mass | -0.0018 | 0.002 | 0.46 |
| Abbreviation: SE: Standard error. | | | |

Supplementary Table 8. MR-PRESSO analyses between exposures and outcomes with outlier-corrected

| Exposure | MR Analysis | Causal Estimate | SD | *p*-value |
| --- | --- | --- | --- | --- |
| Basal metabolic rate | Outlier-corrected | 0.18 | 0.06 | 2.78E-03 |
| Body fat percentage | Outlier-corrected | 0.17 | 0.07 | 2.06E-02 |
| Hip circumference | Outlier-corrected | 0.17 | 0.05 | 1.51E-03 |
| Trunk fat mass | Outlier-corrected | 0.12 | 0.05 | 2.31E-02 |
| Trunk fat percentage | Outlier-corrected | 0.19 | 0.06 | 2.27E-03 |
| Waist circumference | Outlier-corrected | 0.17 | 0.07 | 9.18E-03 |
| Weight | Outlier-corrected | 0.16 | 0.06 | 3.96E-03 |
| Whole body fat mass | Outlier-corrected | 0.17 | 0.05 | 1.37E-03 |
| Whole body fat-free mass | Outlier-corrected | 0.15 | 0.06 | 1.14E-02 |
| Whole body water mass | Outlier-corrected | 0.17 | 0.06 | 5.41E-03 |


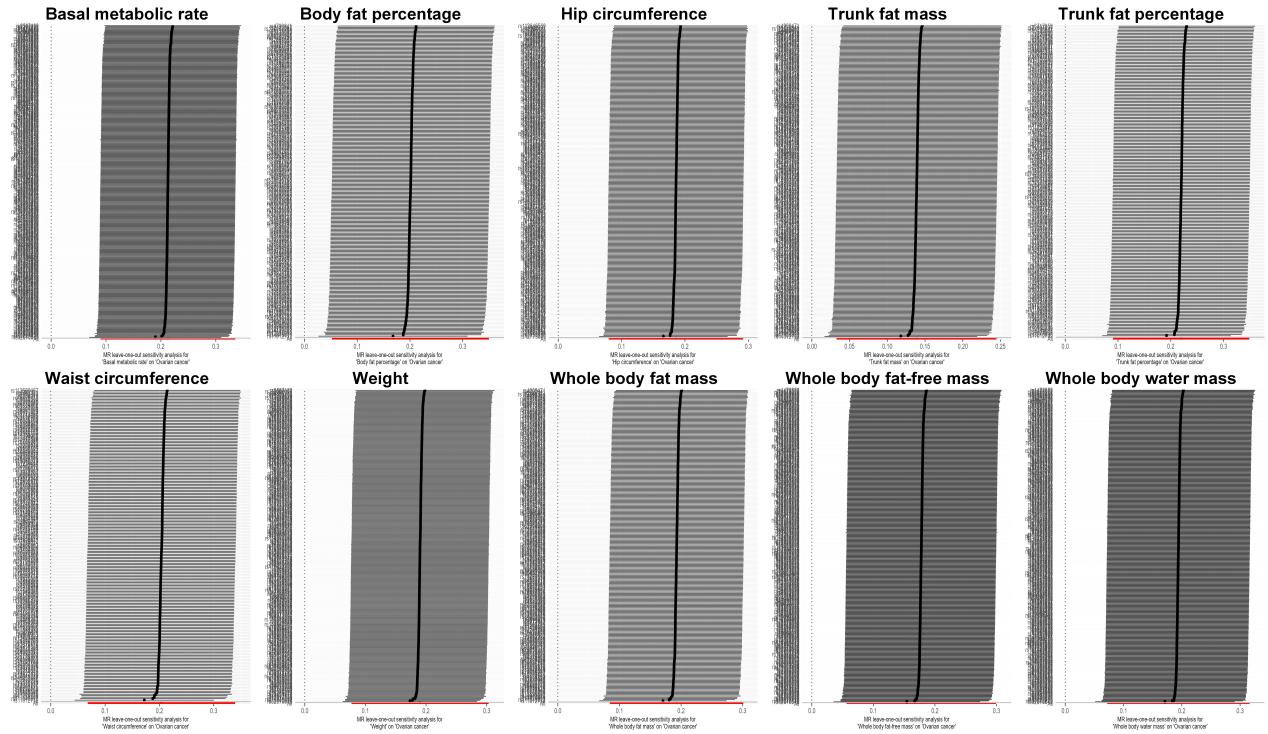


Supplementary Figure 1. Leave-one-out sensitivity analysis using the IVW method to investigate the causal estimates of 10 metabolic factors on ovarian cancer after excluding a particular SNP from the analysis. MR: Mendelian randomization; SNP: single nucleotide polymorphism; IVW: inverse-variance weighted.
